# Glutathione de novo synthesis but not recycling process coordinates with glutamine catabolism to control redox homeostasis and directs murine T cell differentiation

**DOI:** 10.7554/eLife.36158

**Published:** 2018-09-10

**Authors:** Gaojian Lian, JN Rashida Gnanaprakasam, Tingting Wang, Ruohan Wu, Xuyong Chen, Lingling Liu, Yuqing Shen, Mao Yang, Jun Yang, Ying Chen, Vasilis Vasiliou, Teresa A Cassel, Douglas R Green, Yusen Liu, Teresa WM Fan, Ruoning Wang

**Affiliations:** 1 Center for Childhood Cancer and Blood Diseases, Hematology, Oncology and BMThe Research Institute at Nationwide Children's Hospital, Ohio State UniversityColumbusUnited States; 2 Medical Research CenterUniversity of South ChinaHengyang, Hunan ProvinceChina; 3 Department of ImmunologySt. Jude Children’s Research HospitalMemphisUnited States; 4 Department of SurgerySt. Jude Children’s Research HospitalMemphisUnited States; 5 Department of Environmental Health Sciences, Yale School of Public HealthYale UniversityNew HavenUnited States; 6 Department of Toxicology and Cancer BiologyUniversity of KentuckyLexingtonUnited States; 7 Markey Cancer CenterUniversity of KentuckyLexingtonUnited States; 8 Center for Environmental and Systems BiochemistryUniversity of KentuckyLexingtonUnited States; 9 Center for Perinatal ResearchThe Research Institute at Nationwide Children's Hospital, Ohio State UniversityColumbusOhio, United States; University of OxfordUnited Kingdom; Institute of Industrial Science, The University of TokyoJapan

**Keywords:** T cells, ROS, glutathione, activation, differentiation, Mouse

## Abstract

Upon antigen stimulation, T lymphocytes undergo dramatic changes in metabolism to fulfill the bioenergetic, biosynthetic and redox demands of proliferation and differentiation. Glutathione (GSH) plays an essential role in controlling redox balance and cell fate. While GSH can be recycled from Glutathione disulfide (GSSG), the inhibition of this recycling pathway does not impact GSH content and murine T cell fate. By contrast, the inhibition of the de novo synthesis of GSH, by deleting either the catalytic (Gclc) or the modifier (Gclm) subunit of glutamate–cysteine ligase (Gcl), dampens intracellular GSH, increases ROS, and impact T cell differentiation. Moreover, the inhibition of GSH de novo synthesis dampened the pathological progression of experimental autoimmune encephalomyelitis (EAE). We further reveal that glutamine provides essential precursors for GSH biosynthesis. Our findings suggest that glutamine catabolism fuels de novo synthesis of GSH and directs the lineage choice in T cells.

## Introduction

Glutathione (GSH) is the most abundant antioxidant capable of providing reducing equivalents and it also serves as a versatile nucleophilic cofactor in a wide spectrum of metabolic reactions in aerobic organisms (Kosower and Kosower, 1978; Meister, 1982). While some cells are capable of employing extracellular GSH, the utilization of extracellular GSH plays a minor role in regulating GSH homeostasis, since the extracellular levels of GSH are normally three orders of magnitude lower than intracellular GSH concentrations, which are usually in the millimolar range (Kosower and Kosower, 1978; Meister, 1982; Perrone et al., 2005; Hwang et al., 1992; Ganguly et al., 2003; Bennett et al., 2009; Morgan et al., 2011; Park et al., 2016). Hence, cellular GSH content is largely determined by intracellular production through de novo synthesis, a process mediated by two ATP-dependent ligases, glutamate-cysteine ligase (GCL) and glutathione synthase (GS), as well as through regeneration of GSH from GSSG, a process catalyzed by glutathione disulfide reductase (GSR) (Meister, 1982; Lu, 2009). In the process of de novo synthesis, GCL, a heterodimer of a catalytic subunit (GCLC) and a modifier subunit (GCLM), catalyzes the first and rate-limiting step to form the dipeptide γ-glutamylcysteine (γ-GC) from cysteine and glutamate (Franklin et al., 2009; Chen et al., 2005). After the initial step, GS catalyzes the formation of GSH by ligating γ-GC with glycine. As such, GSH synthesis is determined by the availability of its constituent amino acids, cysteine, glycine and glutamate, which intersects with glucose and glutamine metabolic pathways and reflects the overall metabolic status in the cell. In particular, glutamine catabolism may coordinate with de novo GSH synthesis by promoting cysteine uptake and providing glutamate, an immediate product of glutamine after deamination (Altman et al., 2016; Hensley et al., 2013; Gorrini et al., 2013). However, it remains unknown the extent to which de novo synthesis versus recycling of GSSG contributes to GSH homeostasis in T cells and how the perturbation of GSH homeostasis impacts T cell differentiation.

The regulation of metabolic pathways is tightly linked with T cell activation, differentiation, and immune functions (Wang and Green, 2012; Pearce and Pearce, 2013; O'Neill et al., 2016; Patel and Powell, 2017; Ma et al., 2017; Buck et al., 2017; Zeng and Chi, 2017; Finlay and Cantrell, 2011; Weinberg et al., 2015). We and others have shown that activation of T cells leads to a significant enhancement of aerobic glycolysis but a suppression of mitochondria-dependent fatty acid oxidation (FAO) (Wang et al., 2011; Frauwirth et al., 2002; Jacobs et al., 2008; Shi et al., 2011). Following the initial growth stage of T cell activation, FAO fuels and drives induced CD4^+^ regulatory T (Treg) cell differentiation (Shi et al., 2011; Michalek et al., 2011). In contrast, a persistent glycolytic program is engaged not only during the initial growth phase of T cell activation but also throughout the differentiation of other CD4^+^T helper (T_H_) cells and CD8^+^ cytotoxic T (CTL) cells (Shi et al., 2011; Michalek et al., 2011; Finlay et al., 2012). However, oxygen consumption is also dramatically elevated in active T cells since heightened glutamine catabolism via mitochondria-dependent oxidation following T cell activation fuels oxidative phosphorylation (OXPHOS) by providing α-ketoglutarate (α-KG), an anaplerotic substrate of the tricarboxylic acid cycle (TCA cycle) (Wang et al., 2011; Sena et al., 2013; Kamiński et al., 2012). OXPHOS in mitochondria generates ATP through the electron transport chain (ETC) and also produces reactive oxygen species (ROS) as byproducts, rendering mitochondria a major source of intracellular ROS. Superoxide anion (O_2_^•−^), the ‘primary’ ROS derived from mitochondrial ETC, is converted to hydrogen peroxide (H_2_O_2_) by spontaneous and enzymatic processes, whereupon H_2_O_2_ freely diffuses into cytosol and functions as a redox signaling molecule to elicit a diverse array of cellular responses, the spectrum of which depends on the level of ROS (Sena et al., 2013; Kamiński et al., 2012; Murphy, 2009; Schieber and Chandel, 2014). A fine-tuned balance between ROS generation and antioxidant capacity ensures physiological levels of intracellular ROS, which are required for driving essential signaling events to support T cell-mediated immune responses (Weinberg et al., 2015; Murphy and Siegel, 2013; Kamiński et al., 2013; Simeoni et al., 2016; Rashida Gnanaprakasam et al., 2018). Accordingly, oxidative stress, occurring when ROS generation exceeds the capacity of antioxidants, dampens essential cellular processes and functions of T cells. In innate immune cells, ROS are effector molecules that are capable of directly killing pathogens as well as act as redox signaling molecules that modulate a wide range of innate immune responses (Schieber and Chandel, 2014; Mills et al., 2017; Nathan and Cunningham-Bussel, 2013). Accumulating evidence has shown that ROS production is induced following T cell activation and is required for driving T cell activation and proliferation (Sena et al., 2013; Kamiński et al., 2012). Also, T_H_17 cells are more sensitive to the damaging effects of ROS than are T_reg_ cells (Gerriets et al., 2015). A recent study demonstrated a critical role for GSH biosynthesis in fine-tuning this process by maintaining ROS hemostasis and regulating Myc-dependent T cell metabolic reprogramming during T cell activation (Mak et al., 2017; Klein Geltink et al., 2017). Little information exists, however, on whether and how T cell metabolic programs modulate T cell GSH biosynthesis and ROS homeostasis.

Here, we report a critical role for de novo GSH synthesis but not recycling of GSSG in modulating ROS homeostasis and T cell differentiation. Heightened glutamine catabolism during T_H_17 differentiation provides glutamate to support de novo GSH synthesis and suppresses oxidative stress. Genetic ablation of de novo synthesis of GSH but not regeneration of GSH from GSSG leads to the augmentation of ROS, dampening T_H_17 differentiation while enhancing T_reg_ cell differentiation. Moreover, we found that dimethyl fumarate, an FDA approved drug (BG-12/Tecfidera) for multiple sclerosis, suppresses T_H_17 differentiation by augmenting intracellular ROS. Combining pharmacological and genetic approaches, our studies implicate the GSH-ROS axis as a metabolic checkpoint coordinating glutamine catabolism and T cell signaling to direct T cell differentiation.

## Results

### De novo synthesis but not recycling of GSSG is required for producing GSH and suppressing ROS upon TCR stimulation

**Figure 1.**
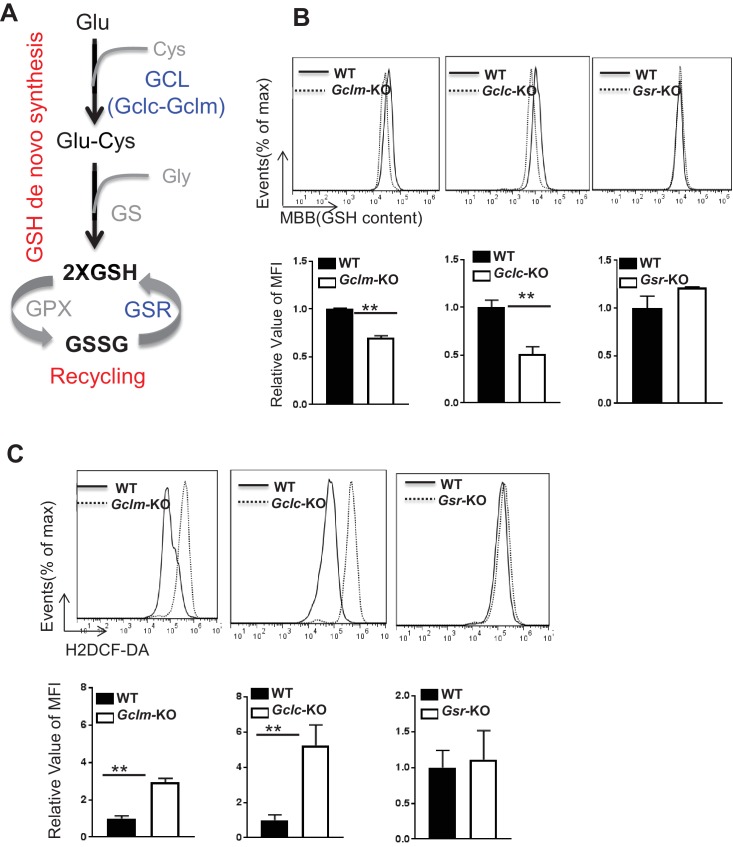
De novo synthesis but not recycling of GSSG is required for producing GSH and fine-tuning ROS upon TCR stimulation. (**A**) Diagram of GSH biosynthesis, with metabolic pathways highlighted in red and enzymes highlighted in blue. (**B**) Naive CD4^+^T cells from WT and *Gclm* KO (left), or WT (*CD4-Cre-, Gclc^fl/fl^*) and *Gclc* KO (*CD4-Cre+, Gclc^fl/fl^*, (middle), or WT and *Gsr* KO (right) were activated by plate-bound anti-CD3 plus anti-CD28 for 24 hr, followed by the measurement of GSH levels. (**C**) Naive CD4^+^T cells from WT and *Gclm* KO (left), or WT (*CD4-Cre-, Gclc^fl/fl^*) and *Gclc* KO (*CD4-Cre+, Gclc^fl/fl^,* (middle), or WT and *Gsr* KO (right) were activated by plate-bound anti-CD3 plus anti-CD28 for 24 hr, followed by the measurement of ROS levels. Data in Figure 1B–C are representative of two independent experiments. Data represent the mean ± S.D. 10.7554/eLife.36158.005Figure 1—source data 1.Source data for B and C.

T cell activation is associated with enhanced GSH and ROS production (Figure 1—figure supplement 1A and B) (Sena et al., 2013; Kamiński et al., 2012; Mak et al., 2017). GSH can be regenerated through recycling of glutathione disulfide (GSSG) or synthesized de novo from glutamate, cysteine and glycine (Figure 1A). GSH regeneration is mediated by glutathione-disulfide reductase (GSR), whereas de novo synthesis is composed of two steps catalyzed by glutamine-cysteine ligase (GCL), a heterodimer of a catalytic subunit (GCLC) and a modulatory subunit (GCLM), and glutathione synthase (GS), respectively (Figure 1A). Thus, we examined the expression of the above key enzymes following T cell activation. Real time quantitative PCR (qPCR) analysis revealed a time-dependent up-regulation of mRNAs encoding these metabolic enzymes in T cells following activation (Figure 1—figure supplement 1C).

**Figure 1—figure supplement 1.**
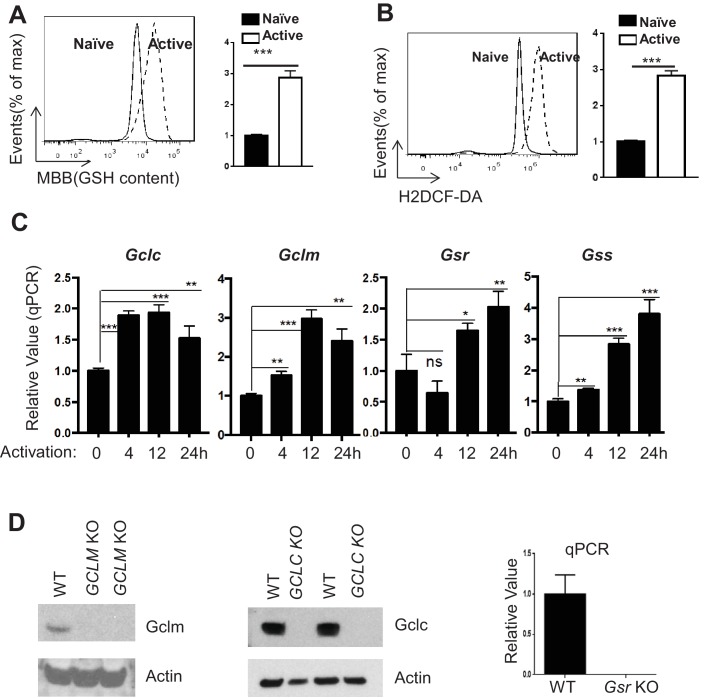
TCR stimulation drives GSH and ROS production in T cells. (**A–B**) Naive CD4 +T cells from C57BL/6 mice were either cultured in the presence of IL-7 (naive) or activated by plate-bound anti-CD3 and anti-CD28 for 24 hr, followed by measuring intracellular GSH (**A**) and ROS (**B**) by FACS. (**C**) RNAs were isolated from naïve or activated T cells for indicated times, and used for real-time qPCR analyses of indicated genes. Expression levels in naive cells were set to 1. (**D**) The protein levels of Gclm (left) or Gclc (middle) in total T cells from mice with indicated genotypes were determined by western blot. RNAs were isolated from WT or *Gsr* KO T cells and used for real-time qPCR analyses of *Gsr* gene. Expression levels in WT sample were set to 1. Data in Figure A-C are representative of two independent experiments. Data are represented the mean ± S.D. 10.7554/eLife.36158.004Figure 1—figure supplement 1—source data 1.Source data for A, B, C and D.

To determine the extent to which de novo synthesis contributes to GSH production and redox homeostasis in T cells, we obtained mouse models with genetic deficiencies in GCL. GCLC possesses all the enzymatic activity, while GCLM functions to optimize the catalytic efficiency of the holoenzyme (Chen et al., 2005). *Gclm* knockout (*Gclm* KO) mice carry the germ-line deletion of *Gclm*, whereas T cell-specific *Gclc* knockout (T cell*-Gclc* KO) mice, generated by crossing *Gclc-floxed* mice with CD4-Cre mice, carry the *Gclc* deletion exclusively in T cells (Chen et al., 2007; Yang et al., 2002). Absent expression of GCLM or GCLC in T cells derived from corresponding animals was confirmed by western blot (Figure 1—figure supplement 1D). Next, we examined the intracellular levels of GSH and ROS of T cells that were stimulated with anti-CD3 plus anti-CD28. Deficiency in GCLC (the catalytic subunit) and, to a lesser extent, deficiency in GCLM (modifier subunit) resulted in reduced intracellular content of GSH (Figure 1B). Consistent with this, we observed increased ROS in *Gclc*- and to a lesser extent in *Gclm*-deficient T cells as compared to WT T cells. (Figure 1C).

We then sought to determine the extent to which recycling of GSSG contributes to GSH production and redox homeostasis in T cells. For this, we obtained mice carrying germ-line deletion of *Gsr (Gsr^-/-^),* the deletion of which was demonstrated by qPCR (Figure 1—figure supplement 1D) (Rogers et al., 2004; Pretsch, 1999; Yan et al., 2012). However, WT and *Gsr*-deficient T cells displayed comparable GSH and ROS levels (Figure 1B and C). These results suggested that de novo synthesis of GSH by the metabolic pathway plays an indispensable role in producing GSH and maintaining redox homeostasis during T cell activation.

### GCLC deficiency but not GCLM or GSR deficiency suppresses T cell activation and proliferation

**Figure 2.**
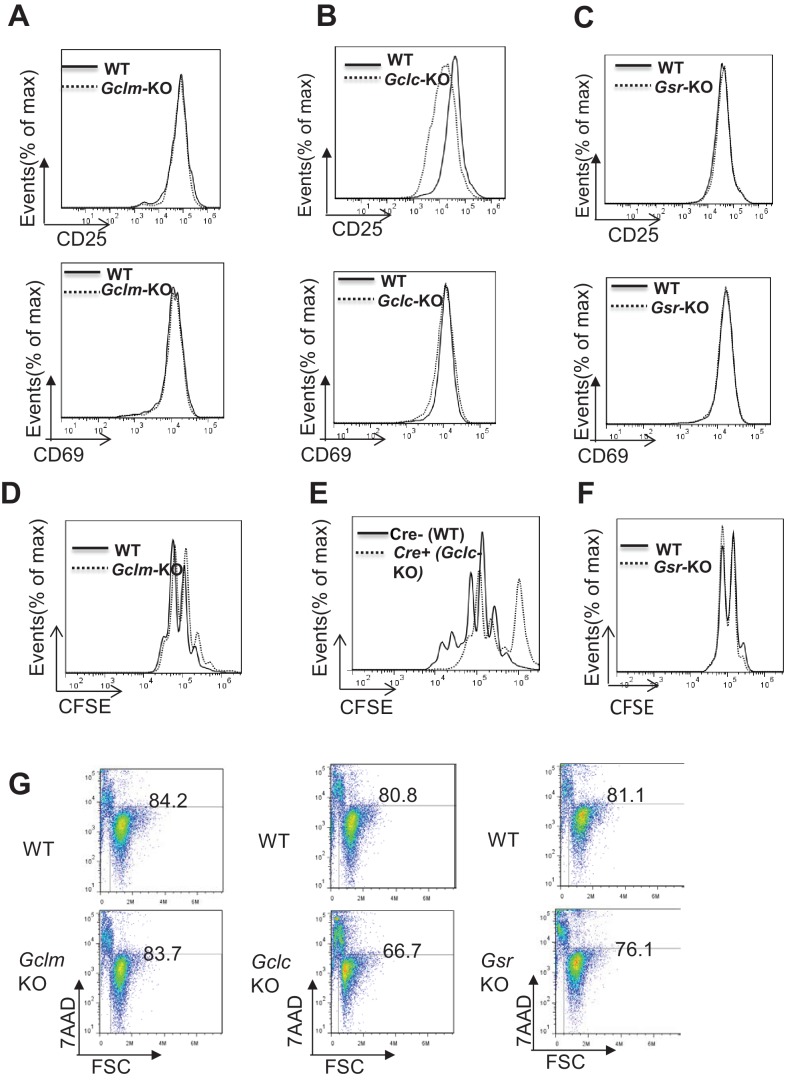
Severe depletion of GSH by blocking de novo synthesis suppresses T cell activation and proliferation. (**A–C**) Naive CD4 +T cells from WT and *Gclm* KO (**A**), or WT (*CD4-Cre-, Gclc^fl/fl^*) and *Gclc* KO (*CD4-Cre+, Gclc^fl/fl^*) (**B**), or WT and *Gsr* KO (**C**) mice were activated by plate-bound anti-CD3 plus anti-CD28 for 24 hr, followed by cell surface expression of CD25 (upper panel) and CD69 (lower panel). (**D–F**) Cell proliferation of active CD4 +T cells (72 hr) with indicated genotypes was determined by CFSE dilution. (**G**) Naive CD4 +T cells isolated from mice with indicated genotypes were activated by plate-bound anti-CD3 and anti-CD28 for 24 hr. Cell viability was determined by FACS. Figure 2A–G are representative of three independent experiments.

*Gclm* KO, T cell*-Gclc* KO and *Gsr* KO mice contained comparable numbers and distribution of thymocytes and peripheral CD4^+^ and CD8^+^ T cells relative to control mice (Figure 2—figure supplement 1A,B,C and D), indicating a largely undisturbed T cell development and distribution after double positive stage in the absence of GSH recycling pathway or the de novo synthesis pathway. A recent study has showed that Gclc deficiency suppressed T cell activation and proliferation, demonstrating a critical role of GCLC in regulating T cell activation (Mak et al., 2017; Klein Geltink et al., 2017). This is consistent with the severe GSH depletion and ROS induction in *Gclc*-deficient active T cells (Figure 1B and C). Our results further suggested that GSR is dispensable and GCLM only play a minor role in modulating GSH production and ROS homeostasis in active T cells (Figure 1B and C). Next, we sought to differentiate the impact of GSH recycling pathway and de novo synthesis pathway on T cell activation and proliferation. While both Gclc and Gclm deficiency caused a reduction of GSH and induction of ROS, albeit to different degrees (Figure 1B and C), Gclc but not Gclm deficiency resulted in an impairment of cell viability, appearance of the activation marker CD25, and activation-induced cell proliferation (Figure 2A, B, D, E and G). This activation and proliferation defect is consistent with a recent study showing that GCLC is required for T cell activation (Mak et al., 2017). Previous studies have shown that GSH depletion caused inactivation of glutathione peroxidase 4 (GPX4), and consequentially led to iron-dependent accumulation of lipid peroxidation and a form of cell necrosis referred as ferroptosis (Stockwell et al., 2017). Our data suggested that a moderate reduced cell viability in *Gclc*-deficient T cells is likely due to ferroptosis in the context of GSH depletion (Figure 2G). Our results suggested that a severe depletion of GSH and induction of ROS caused by Gclc deficiency significantly impaired T cell activation and proliferation, however, active T cell could tolerate and cope with a moderate depletion of GSH and induction of ROS caused by Gclm deficiency.

**Figure 2—figure supplement 1.**
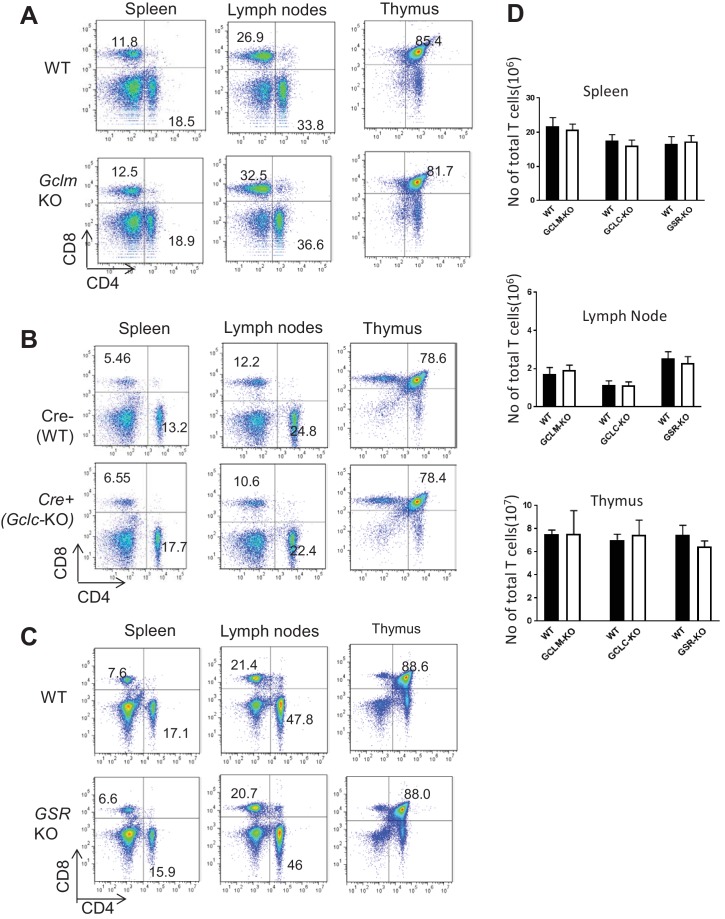
Severe depletion of GSH by blocking de novo synthesis suppresses T cell activation and proliferation. (**A–C**) Distribution of CD4+ and CD8+ cells in the spleen, lymph nodes or thymus from mice with indicated genotypes were determined by FACS. Figure 2A -D are representative of two independent experiments (**D**) Bar graph represents the number of T cells calculated from total splenocytes, lymph node cells and thymocytes. 10.7554/eLife.36158.008Figure 2—figure supplement 1—source data 1.Source data for A, B, C and D.

Consistent with its dispensable role in GSH production in active T cells (Figure 1B and C), Gsr deficiency did not lead to any impairment of T cell viability, activation marker CD25 and proliferation (Figures 2C, F and G). Notably, none of the above genetic deficiencies compromised the early activation marker CD69 (Figure 2A–C). Our results suggest that GSH recycling pathway is dispensable in regulating T cell activation and proliferation. However, the recycling of GSSG to GSH, which is not a parallel pathway for GSH production, plays a critical role in maintaining redox homeostasis when the ratio of GSSG:GSH reaches certain threshold. While our data suggest that Gsr mediated recycling of GSSG is not required for T cell activation and proliferation, we do not have evidence showing that GSSG is significantly accumulated during T cell activation and proliferation. Therefore, a dispensable role of Gsr in maintaining redox homeostasis during T cell activation and proliferation may represent a context-dependent interpretation.

### Ablation of de novo synthesis but not recycling of GSSG reciprocally alters T_H_17 and iT_reg_ cell differentiation

**Figure 3.**
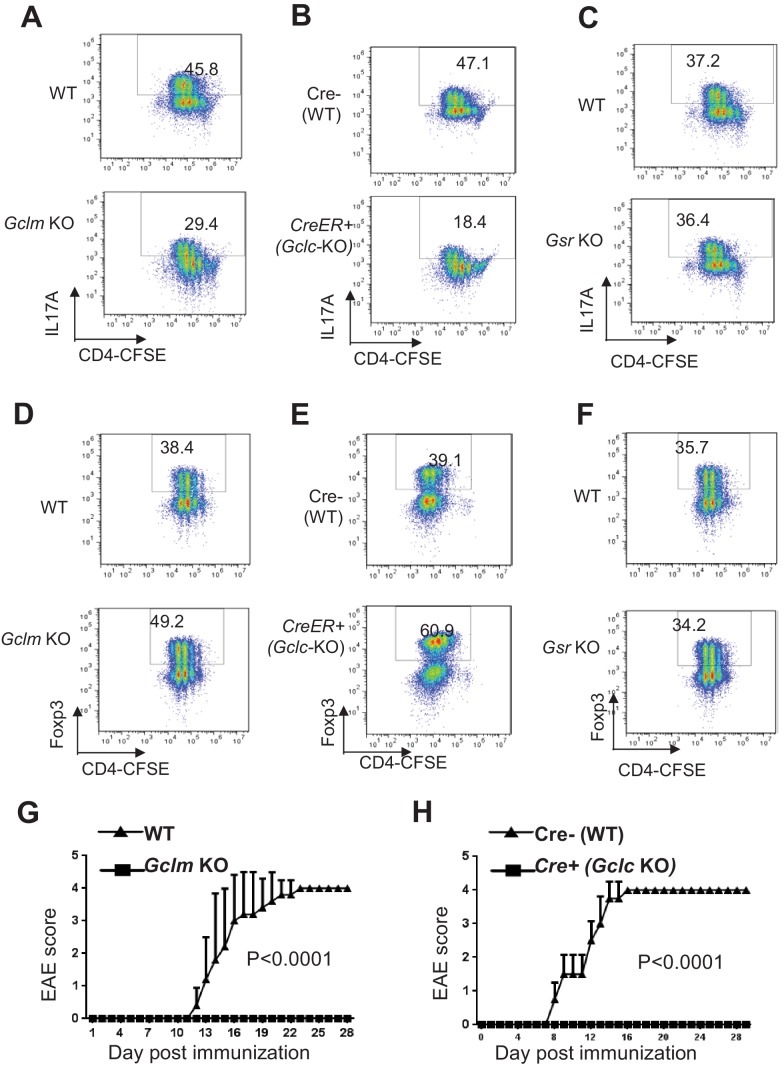
Ablation of de novo synthesis but not recycling of GSSG reciprocally alters T_H_17 and iTreg cell differentiation. (**A–F**) Naive CD4+ T cells from WT and *Gclm* KO, or WT (*Cre-,) and CreER*+ (*Gclc*-KO- in the presence of 100 nM of 4-hydroxytamoxifen (4OHT)), or WT and *Gsr* KO mice were stained with 4 µm CFSE and differentiated under T_H_17 or iTreg -inducing conditions for 5 days, followed by intracellular staining of IL-17 and Foxp3. (**G–H**) mice with indicated genotypes were immunized with MOG to induce EAE and pathological progressions were scored daily. Data in Figure 4A–H are representative of two-three independent experiments. 10.7554/eLife.36158.012Figure 3—source data 1.Source data for G and H.

Following the initial growth stage of cell activation, proliferating CD4^+^ T cells can differentiate into various functional subsets including inflammatory T_H_17 and Foxp3-expressing regulatory T cells (T_reg_ cells), which are two closely related subsets with distinct functions. Having found a role for de novo synthesis of GSH in modulating GSH and ROS homeostasis during T cell activation, we next assessed the functional requirement for the de novo synthesis pathway of GSH in T cell differentiation. Naive T cells were differentiated under T_H_17 or iT_reg_ conditions. As compared to control WT cells, *Gclm* KO cells exhibited reduced IL-17^+^ and increased Foxp3^+^ cells (Figure 3A and D). Given that a similar degree of proliferation was observed between WT and *Gclm* KO CD4^+^ T cells (Figure 3A and D), deregulation of cell differentiation in *Gclm* KO was largely independent of cell expansion. To bypass the effect of Gclc deficiency on T cell activation and proliferation (Figure 2B and E), we generated a mouse model carrying a conditional *Gclc* allele (*Gclc*^*flox/flox*^) and a tamoxifen-induced Cre recombinase (CreERT2) transgene (Ryan et al., 2000; de Luca et al., 2005), which allowed us to delete *Gclc* flox alleles in an acute manner. For this, we polarized T cells in the absence (WT) or in the presence (KO) of 4-Hydroxytamoxifen (4OHT) (Figure 3—figure supplement 1A). As compared with WT cells, acute deletion of Gclc bypassed its required for cell proliferation, as revealed by a comparable CFSE dilution (Figure 3B and E), but nevertheless resulted in a reduction in the generation of IL-17^+^ T cells and an induction in the generation of Foxp3^+^ cells. Using this genetic model, we have therefore differentiated the role of GCLC in early T cell activation from its role in driving T_H_17 cell differentiation. In contrast to the effects of ablation of *Gclm* and *Gclc*, *Gsr* KO and WT CD4^+^ T cells displayed a similar degree of T_H_17 and iT_reg_ differentiation, indicating a dispensable role for recycling GSSG in regulating T cell differentiation (Figure 3C and F). Next, we tested whether inhibiting ROS generation in T cells that have defects on the de novo synthesis of GSH would restore T_H_17 differentiation. The addition of N-acetyl-L-cysteine (NAC), a reagent often used to scavenge ROS, restored T_H_17 cell differentiation in *Gclc^-/-^* T cells (Figure 3—figure supplement 1B). While NAC is frequently considered a source of cysteine for synthesis of GSH, while other studies have shown that NAC displayed reducing properties through its thiol-disulfide exchange activity and could directly scavenge free radicals (Ates et al., 2008; Agnihotri and Mishra, 2009; Zafarullah et al., 2003; Cotgreave, 1997). Consistent with these, our result suggests that NAC scavenges ROS independently of GSH synthesis (Figure 3—figure supplement 1C and D) and therefore restores T_H_17 cell differentiation in *Gclc^-/-^* T cells. To further evaluate the role of the GSH de novo synthesis pathway in T_H_17-driven EAE in vivo, we immunized mice with the myelin oligodendrocyte glycoprotein (MOG)_35-55_ antigen. Genetic ablation of GCLM or GCLC conferred a protection against disease progression (Figure 3G and H). Furthermore, histological assessment revealed a similar degree of ablation of T cell infiltration in *Gclm* KO and T cell*-Gclc* KO animals compared to WT animals (Figure 3—figure supplement 1E). However, a different degree of ablation of macrophage infiltration in *Gclm* KO and T cell*-Gclc* KO animals compared to WT animals was observed and likely reflected a different level of inflammation in these experimental animals (Figure 3—figure supplement 1E). In contrast to T cell*-Gclc* KO animals, GCLM is also deleted in macrophages in *Gclm* KO animal (a germline KO model). Therefore, we could not exclude the possibility that Gclm deficiency in macrophages might affect macrophage infiltration in our result. Collectively, our studies suggested that de novo synthesis of GSH is required for T_H_17 development in vitro and T_H_17-driven CNS inflammation in vivo.

**Figure 3—figure supplement 1.**
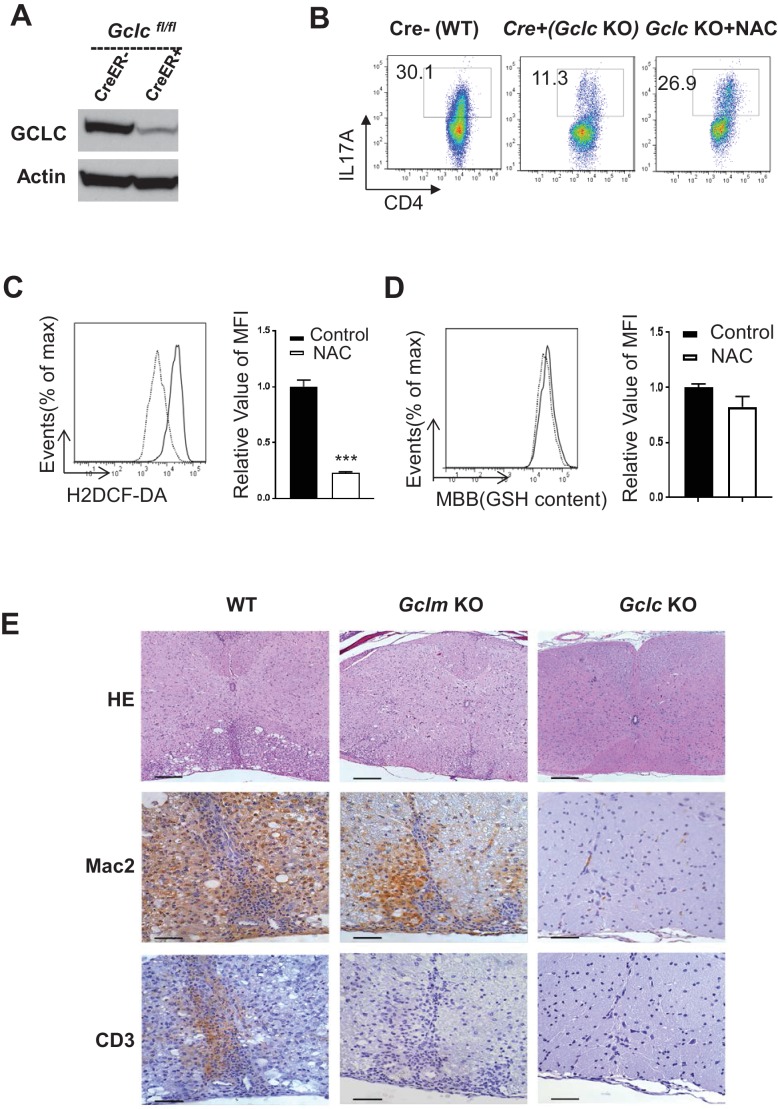
Ablation of de novo synthesis but not recycling of GSSG reciprocally alters TH17 and iTreg cell differentiation. (**A**) The protein levels of Gclc in cells collected two days following 4OHT treatment were determined by western blot. (**B**) Naïve CD4+ T cells from WT (*CD4-Cre-, Gclcfl/fl*) and *Gclc KO (CD4-Cre+, Gclcfl/fl)* mice were differentiated under TH17-inducing conditions with or without 5 mM N-acetyl cysteine (NAC) for 5 days, followed by intracellular staining of IL-17. (**C**) Naive CD4+ T cells were activated for 24 hr with or without 5 mM NAC, followed by measuring ROS and (**D**) GSH by FACS. (**E**) WT, *Gclm-/-* (KO) and *CD4-Cre, Gclc^fl/fl^* (KO) mice were immunized with MOG/CFA. After 20 days, cervical spinal cord sections were analyzed by H and E (bars, 200 μm) and anti-Mac2 and anti-CD3 immunohistochemistry (bars, 50 μm).Data are representative of two independent trials. Data in Figure A-D are representative of two-three independent experiments. 10.7554/eLife.36158.011Figure 3—figure supplement 1—source data 1.Source data for C and D.

### T_H_17 and iT_reg_ cells display different degrees of oxidative stress

**Figure 4.**
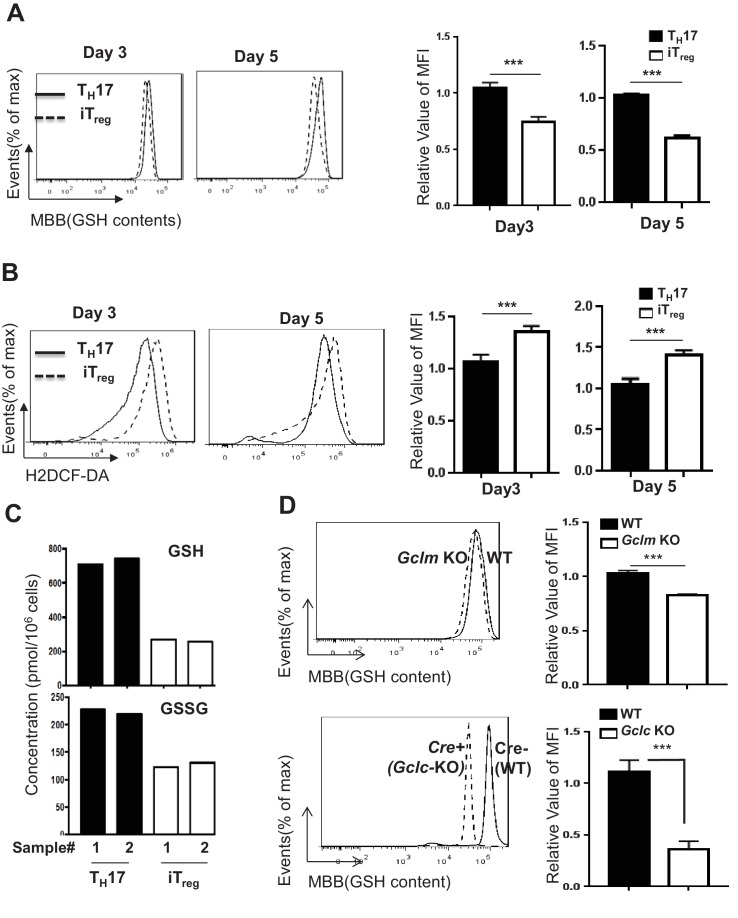
T_H_17 cells preferentially maintain higher level of GSH than iT_reg_ cells. (**A–B**) Naive CD4^+^ T cells from C57BL/6 mice were differentiated under iTreg or T_H_17–inducing conditions and cells were collected at indicated times, followed by measuring intracellular GSH (**A**) and ROS (**B**) by FACS. (**C**) Naive CD4^+^ T cells from C57BL/6 mice were differentiated under T_H_17 or iT_reg_–inducing conditions for 5 days. The intracellular levels of GSH and GSSG were determined by mass spectrometry. (**D**) Naive CD4^+^T cells from WT and *Gclm* KO (top) or WT (*CD4-Cre-, Gclc^fl/fl^*) and *Gclc* KO (*CD4-Cre+, Gclc^fl/fl^*, (bottom) were differentiated under T_H_17-inducing conditions for 5 days, followed by the measurement of GSH levels. Data in Figure 4A–D are representative of three independent experiments. Data represent the mean ± S.D. 10.7554/eLife.36158.016Figure 4—source data 1.Source data for A, B, C and D.

Accumulating evidence has shown that each subset of T cells engages unique metabolic pathways to fulfill its metabolic demands (Shi et al., 2011; Michalek et al., 2011; Gerriets et al., 2015). We envisioned that the differential engagement of metabolic pathways would differentially impact GSH biosynthesis and cellular oxidative stress in T_H_17 and iT_reg_ cells. For this, we activated naive CD4^+^ T cells under T_H_17 or iT_reg_ polarizing conditions in vitro, and examined the intracellular levels of GSH and ROS at day 3 and day 5. T_H_17 cells displayed a higher level of intracellular GSH than iT_reg_ cells. In contrast, the level of ROS was lower in T_H_17 cells compared to iT_reg_ cells (Figure 4A and B). In addition, T_H_17 cells displayed a higher level of intracellular GSH and GSSG than iT_reg_ cells, as revealed by mass spectrometry (Figure 4C). A key cellular mechanism in defending against oxidative stress is through activation of nuclear factor erythroid 2-related factor 2 (NRF2), which controls the expression of genes involved in producing, regenerating, and utilizing GSH. NRF2 also controls other antioxidant pathways that regulate thioredoxin (*TXN*), NADPH generation and iron sequestration (Jaramillo and Zhang, 2013; Sporn and Liby, 2012; Ma, 2013; Motohashi and Yamamoto, 2004). Consistent with increased levels of GSH and decreased levels of ROS in T_H_17 cells, qPCR analysis revealed a time-dependent up-regulation of mRNAs encoding NRF2 and its target genes, including glucose-6-phosphate dehydrogenase (*G6PD*), *TXN*, thioredoxin reductase 1 (*Txnrd1*), CD44, heme oxygenase-1 (*Hmox1*), NAD(P)H quinone dehydrogenase 1 (*Nqo1*) and glutathione synthetase (*Gss*) (Figure 4—figure supplement 1A). These results suggested that T_H_17 cells preferentially maintain a low degree of oxidative stress by a tight regulation of GSH synthesis and ROS homeostasis.

**Figure 4—figure supplement 1.**
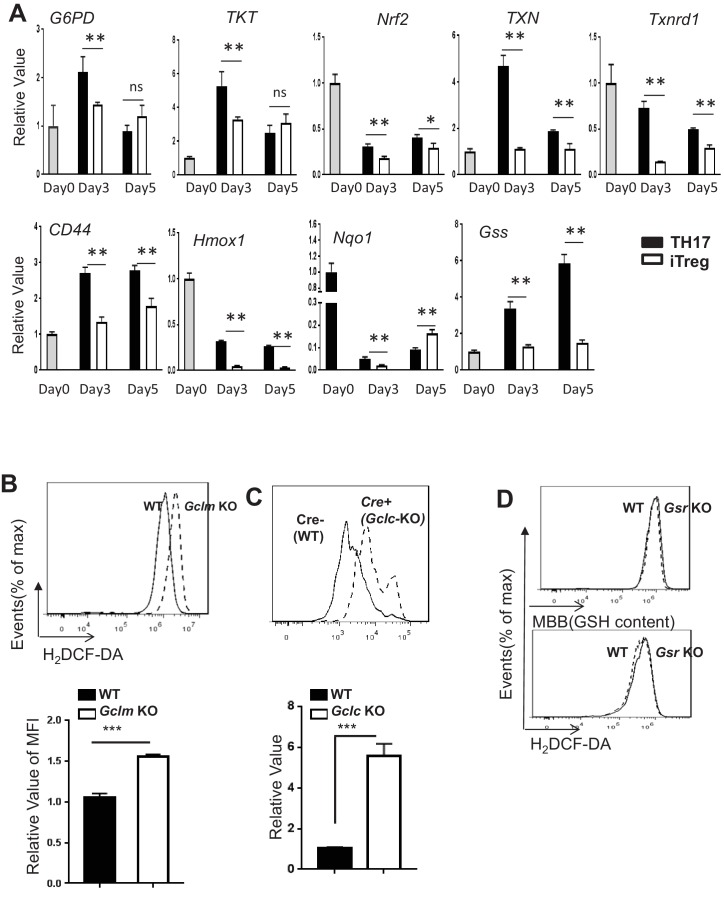
De novo synthesis but not recycling of GSSG is required for providing GSH and suppressing ROS during TH17 cell differentiation. (**A**) RNAs were isolated from cells differentiated under TH17 or iTreg-inducing conditions for indicated times, and used for real-time qPCR analyses of indicated genes. Expression levels in day 0 were set to 1 (**B–D**) Naive CD4+ T cells from WT and *Gclm* KO, or WT (*CD4-Cre-, Gclc^fl/fl^*) and *Gclc* KO (*CD4-Cre+, Gclc^fl/fl^*^),^ or WT and *Gsr* KO mice were differentiated under TH17-inducing conditions for 5 days, followed by measuring ROS by FACS. Data in Figure B-D are representative of two independent experiments. Data represent the mean ±S.D. 10.7554/eLife.36158.015Figure 4—figure supplement 1—source data 1.Source data for A, B and C.

### De novo synthesis but not recycling of GSSG is required for producing GSH and suppressing ROS during T_H_17 cell differentiation

The observation that T_H_17 cells displayed increased GSH content and decreased levels of ROS than iT_reg_ cells supported the role for GSH in directing T_H_17 cell differentiation (Figure 4A and B). Next, we sought to determine how two GSH synthetic pathways are engaged and impact on GSH and ROS homeostasis during T_H_17 cell differentiation. For this, we purified naive T cells and differentiated them under T_H_17-polarizing conditions. Deficiency in Gclc and, to a lesser extent, deficiency in Gclm resulted in reduced intracellular content of GSH (Figure 4D). Consistent with this, we observed increased ROS in *Gclc*- and *Gclm*-deficient T cells as compared to WT T cells (Figure 4—figure supplement 1B and C). By contrast, WT and *Gsr*-deficient T cells displayed comparable GSH sand ROS levels (Figure 4—figure supplement 1D). These results indicated that the preferential requirement for de novo synthesis of GSH during the initial T cell activation stage is extended to the later T cell differentiation stage. However, the recycling pathway is dispensable in producing GSH and maintaining redox homeostasis during T_H_17 differentiation.

### Pharmacological augmentation of ROS reciprocally modulates T_H_17 and iT_reg_ cell differentiation

**Figure 5.**
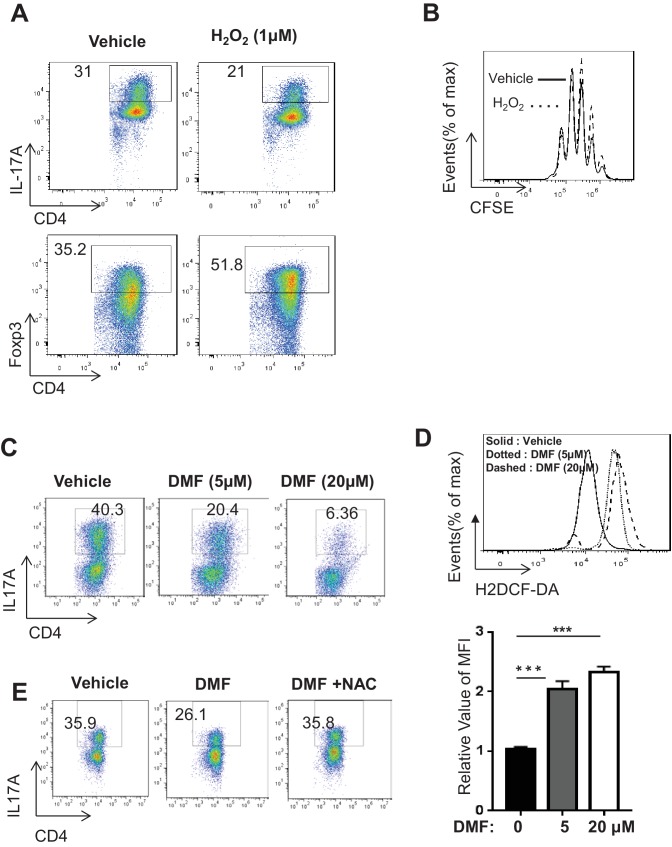
DMF suppresses T_H_17 differentiation by augmenting ROS generation. (**A**) Naive CD4^+^ T cells from C57BL/6 mice were differentiated under T_H_17 or iT_reg_-inducing conditions with or without H_2_O_2_ (1 µM) for 5 days, followed by intracellular staining of IL-17 and Foxp3. (**B**) Cell proliferation of active CD4^+^ T cells (72 hr) with or without H_2_O_2_ (1 µM) was determined as CFSE dilution. (**C–D**) Naive CD4^+^ T cells from C57BL/6 mice were differentiated under T_H_17-inducing conditions with indicated dose of DMF for 5 days, followed by intracellular staining of IL-17 (**C**) and ROS (**D**). (**E**) Naive CD4^+^ T cells from C57BL/6 mice were differentiated under T_H_17-inducing conditions with indicated treatment for 5 days, followed by intracellular staining of IL-17. Data in Figure 5 are representative of two-three independent experiments. Data represent the mean ±S.D. 10.7554/eLife.36158.019Figure 5—source data 1.Source data for D.

We next asked whether shifting the redox balance towards an oxidative state would perturb T cell differentiation and represent a novel therapeutic strategy for T cell-driven autoimmunity. For this, we activated naive CD4^+^ T cells under T_H_17 or iT_reg_ polarizing conditions in the presence or absence of H_2_O_2_. Addition of 1 μM H_2_O_2_ did not impact cell proliferation but reciprocally reduced T_H_17 and enhanced iT_reg_ cell differentiation (Figure 5A and B), indicating that its effect on differentiation was largely independent of expansion.

**Figure 5—figure supplement 1.**
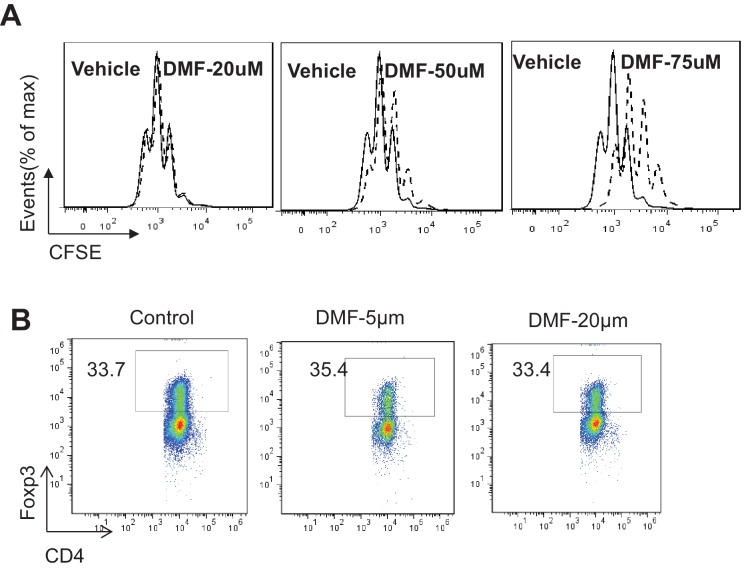
DMF suppresses T_H_17 differentiation through augmenting ROS generation. (**A**) Cell proliferation of active CD4 +T cells (72 hr) with indicated treatments was determined as CFSE dilution (**B**). Naive CD4 +T cells from C57BL/6 mice were differentiated with 5 and 20 µM of DMF under iTreg cell -inducing conditions for 5 days, followed by intracellular staining of Foxp3.

The observation of H_2_O_2_-dependent suppression of T_H_17 differentiation prompted us to explore pharmacologic approaches that could augment ROS production in T cells. Dimethyl fumarate (DMF), the key active ingredient of BG-12/TECFIDERA and FUMADERM, has been approved in many countries for treating autoimmune diseases including multiple sclerosis (MS) and psoriasis, both of which are associated with pathogenic T_H_17 cells (Study group et al., 2014; Mrowietz et al., 2007; DEFINE Study Investigators et al., 2012; CONFIRM Study Investigators et al., 2012). However, the cellular and molecular mechanisms underlying the therapeutic efficacy of DMF have not been fully elucidated (Kees, 2013). Previous studies have implicated DMF in regulating the cellular activities of dendritic cells (DCs), endothelial cells, and neurons through various mechanisms (Blewett et al., 2016; Schulze-Topphoff et al., 2016; Wang et al., 2015; Chen et al., 2014; Peng et al., 2012; Linker and Gold, 2013; Duffy et al., 1998; Gill and Kolson, 2013). The electrophilic nature of DMF allows it to bind and deplete intracellular GSH (Zheng et al., 2015; Sullivan et al., 2013). We therefore hypothesized that DMF may induce oxidative stress and affect T cell differentiation. To test this, we activated naive CD4^+^ T cells under T_H_17 polarizing conditions in the presence of a range of DMF doses based on previous reports that were designed to investigate the effect of DMF on other cell types (Peng et al., 2012; Linker and Gold, 2013; Duffy et al., 1998). DMF treatment displayed a dosage-dependent suppression of T_H_17 cell differentiation (Figure 5C). Although the addition of 75 μM DMF inhibited T cell proliferation, at the lower dose of DMF used in this study (20–50 μM), we observed minimal inhibitory effects of DMF on cell proliferation (Figure 5—figure supplement 1A), indicating that DMF-mediated suppression on T_H_17 differentiation was largely independent of cell proliferation. Moreover, DMF treatments induced ROS production in T_H_17 cells (Figure 5D). Consistent with the idea that DMF suppresses T_H_17 cell differentiation through the induction of oxidative stress, the addition of N-acetyl-L-cysteine (NAC) restored T_H_17 cell differentiation in the presence of DMF (Figure 5E). Since H_2_O_2_ treatment reciprocally reduced T_H_17 and enhanced iT_reg_ cell differentiation (Figure 5A and B), we next assessed the effect of DMF on iT_reg_ cell differentiation. However, the doses of DMF (5 and 20 μM) that could suppress T_H_17 differentiation failed to affect iT_reg_ cell differentiation in vitro (Figure 5—figure supplement 1B). As such, our data suggested that DMF may partially exert its immunomodulatory action through the augmentation of oxidative stress and suppressing T_H_17 differentiation. Along with the possibility that DMF-derived fumarate interferes with TCA cycle intermediate metabolite pool, other mechanisms may also contribute to its immunomodulatory functions (Blewett et al., 2016; Schulze-Topphoff et al., 2016; Wang et al., 2015; Chen et al., 2014; Peng et al., 2012; Linker and Gold, 2013; Duffy et al., 1998; Gill and Kolson, 2013).

### Glutamate that fuels GSH de novo synthesis is partially derived from glutamine during T_H_17 cell differentiation

**Figure 6.**
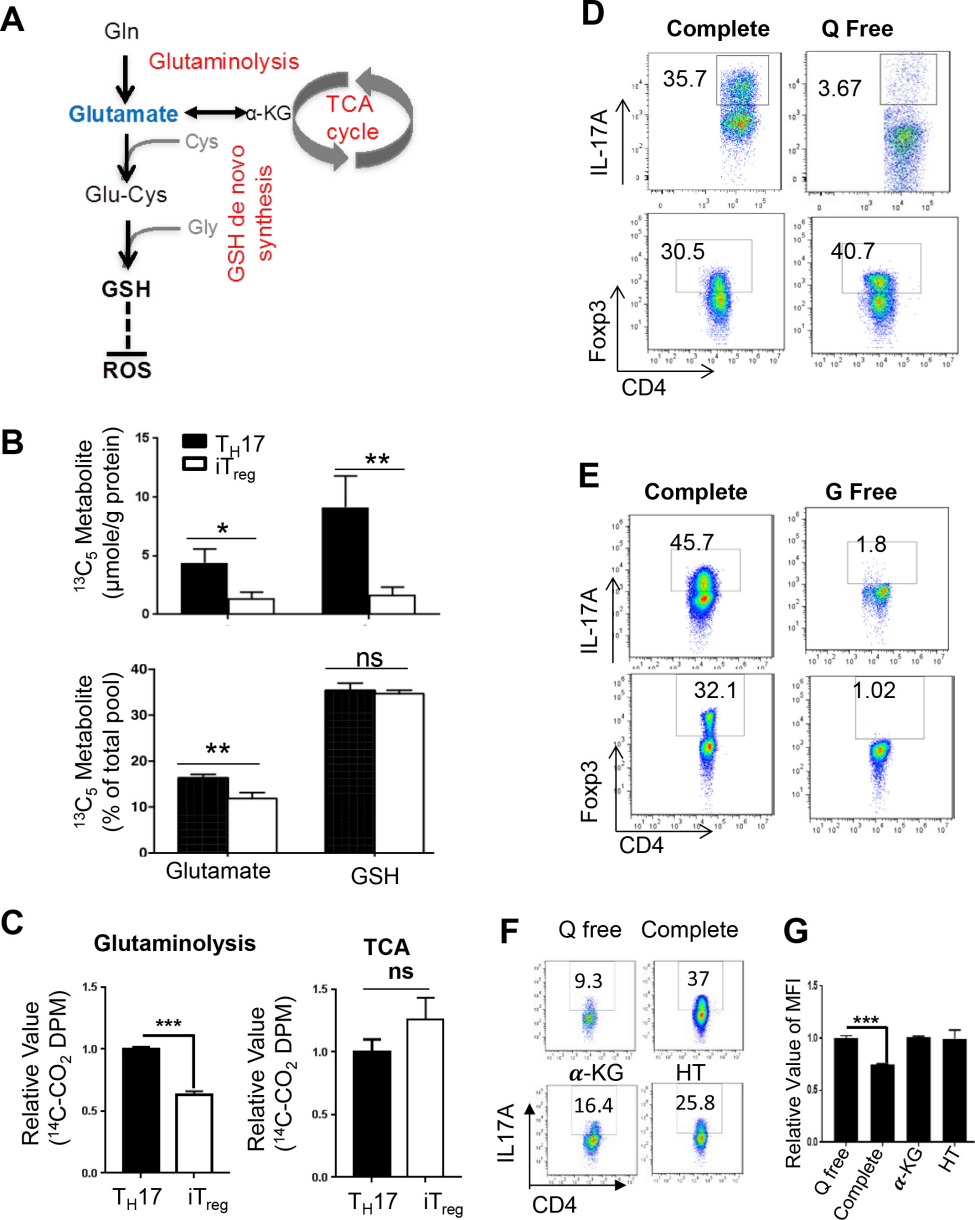
Glutamate that fuels GSH de novo synthesis is partially derived from glutamine in T cells. (**A**) Diagram of metabolic steps linked to the GSH production, with metabolic pathways highlighted in red. (**B**) Naive CD4^+^ T cells from C57BL/6 mice were differentiated under T_H_17 and iT_reg_–inducing conditions for 5 days, followed by culturing in media containing ^13^C_5_^15^N_2_-glutamine. The intracellular levels of Glutamate and GSH including ^13^C-, ^13^C,^15^N-, and ^12^C-unlabeled forms were determined by IC-UHRFTMS. (**C**) Naive CD4^+^ T cells from C57BL/6 mice were differentiated under T_H_17 or iT_reg_ cell–inducing conditions for 5 days, were used for measuring the generation of ^14^CO2 from [U-^14^C]-glutamine (glutaminolysis), from [2-^14^C]-pyrvuate (TCA). (**D–E**) Naive CD4^+^ T cells from C57BL/6 mice were differentiated in completed, glutamine-free (Q free) or glucose-free (G free) medium under T_H_17 or iT_reg_ cell-inducing conditions for 5 days, followed by intracellular staining of IL-17 and Foxp3. (**F**) Naive CD4^+^ T cells from C57BL/6 mice were activated in complete medium for 24 hr and cells were washed with PBS and switch to conditional medium in presence or absence of glutamine, 3 mM αKG or 100 μM hypoxanthine and 16 μM thymidine (HT) for 5 days followed by intracellular staining of IL-17 and (**G**) and ROS. Data represent the mean ±S.D. 10.7554/eLife.36158.024Figure 6—source data 1.Source data for B, C and G.

Upon activation, a metabolic reprogramming is required for directing nutrients to meet the bioenergetic, biosynthetic, and redox demands, which prepares T cells for immune defense and regulation. We and others have shown that T cell metabolism changes from relying on FAO and some mitochondria-dependent glucose oxidation to engaging robust aerobic glycolysis and glutaminolysis (Wang et al., 2011; Frauwirth et al., 2002; Jacobs et al., 2008; Gerriets and Rathmell, 2012; Pearce et al., 2009). Glutamine catabolism not only fuels mitochondrial ATP production through the TCA cycle but also provides metabolic precursors for multiple biosynthetic pathways, including synthesis of glutathione (GSH), an essential cellular antioxidant system to maintain redox homeostasis during T cell activation (Altman et al., 2016; Hensley et al., 2013; Gorrini et al., 2013; Mak et al., 2017). The de novo synthesis of GSH requires glycine, cysteine and glutamate as metabolic precursors. We envisioned that glutamine-derived glutamate partially fulfils the requirement of de novo synthesis of GSH in T_H_17 cells (Figure 6A). To test this hypothesis, we followed U-^13^C,^15^N labeled glutamine incorporation into glutamate and GSH. The ^13^C_5_ isotopologues (generated via a direct Glutamine to Glutmate conversion) represented a significant fraction of the total glutamate and GSH pool in both T_H_17 and iT_reg_ cells, however, the absolute quantity of ^13^C_5_-glutamate and ^13^C_5_-GSH is higher in T_H_17 cells than in iT_reg_ cells (Figure 6B). These results suggested that T_H_17 cells could uptake more glutamine and/or produce more glutamate for GSH synthesis than iT_reg_ cells. In accord with this data, glutamine starvation reduced GSH content in a time-dependent manner in T_H_17 cells (Figure 6—figure supplement 1A). In addition to support GSH biosynthesis, glutamine-derived glutamate can also feed into the TCA cycle. We therefore utilized radiochemical-based approaches to assess activities of glutamine oxidation through the TCA cycle in T_H_17 and iT_reg_ cells. T_H_17 cells displayed higher glutamine oxidation activity, indicated by ^14^CO_2_ release from [U-^14^C]-glutamine, than did iT_reg_ cells (Figure 6C). However, mitochondria-dependent pyruvate oxidation through the TCA cycle, indicated by ^14^CO_2_ release from [2-^14^C]-pyruvate, was comparable between the two functional subsets (Figure 6C). These data further suggested that the overall uptake and consumption of glutamine, including oxidation of glutamate through the TCA cycle and utilization of glutamate for GSH biosynthesis is enhanced in T_H_17 comparing to iT_reg_ cells. Also, qPCR analyses revealed marked upregulation of genes encoding various molecules involved in glutamine catabolism and GSH metabolism in T_H_17 compared to iT_reg_ cells (Figure 6—figure supplement 1B and C). Consistent with this, deprivation of glutamine significantly suppressed T_H_17 but moderately enhanced iT_reg_ cell differentiation (Figure 6D), while both T_H_17 and iT_reg_ differentiation required glucose (Figure 6E). Next, we asked if pharmacological inhibition of the rate-limiting glutaminolyic enzyme glutaminase (Gls) could impact T cell differentiation. Two Gls1 specific inhibitors, bis-2-(5-phenylacetamido-1,2,4-thiadiazol-2-yl) ethyl sulfide (BPTES) and CB-839 (Wang et al., 2010; Le et al., 2012), slightly enhanced IL-17 expression (Figure 6—figure supplement 1D). However, 6-diazo-5-oxo-l-norleucine (DON), an analog of glutamine with broad inhibitory effects glutamine utilizing enzymes (Pinkus, 1977; Shapiro et al., 1979), skewed away T cells from T_H_17 toward iT_reg_ differentiation (Figure 6—figure supplement 1D). In addition, DON but not BPTES and CB-839 significantly enhanced ROS production under T_H_17-polarizing condition (Figure 6—figure supplement 1E). These results suggested that other glutamine utilizing enzymes including glutamine-dependent amidotransferase and Gls2, the latter of which has been shown to be induced upon T cell activation (Wang et al., 2011; Zalkin and Smith, 1998; Massière and Badet-Denisot, 1998).Glutamine catabolism is not only coupled to the de novo synthesis of GSH, but also generates the anaplerotic substrate, α-ketoglutarate (α-KG), and substrates for nucleotide biosynthesis(Altman et al., 2016). A previous study demonstrated that glutamine deprivation enhances iTreg differentiation and addition of α-KG could compromise such effect (Klysz et al., 2015). In line with this report, addition of either hypoxanthine and thymidine (HT) or α-KG was able to partially rescue T_H_17 differentiation in glutamine-free condition without impacting ROS level (Figure 6F and G). While NAC treatment is sufficient to suppress ROS production, it was incapable of rescuing T_H_17 differentiation (Figure 6—figure supplement 1G). However, the combination of HT and NAC led to more differentiated T_H_17 cells and lower level of ROS comparing to HT treatment alone in the absence of glutamine (Figure 6—figure supplement 1F). Taken together, our studies suggest that glutamine catabolism directs the lineage choices between T_H_17 and iT_reg_ cells through supporting T cell proliferation by providing biosynthetic precursors. In addition, glutamine-derived glutamate provides a key substrate for the de novo synthesis of GSH, modulates ROS signaling, and may also impact T cell differentiation (Figure 6—figure supplement 2).

**Figure 6—figure supplement 1.**
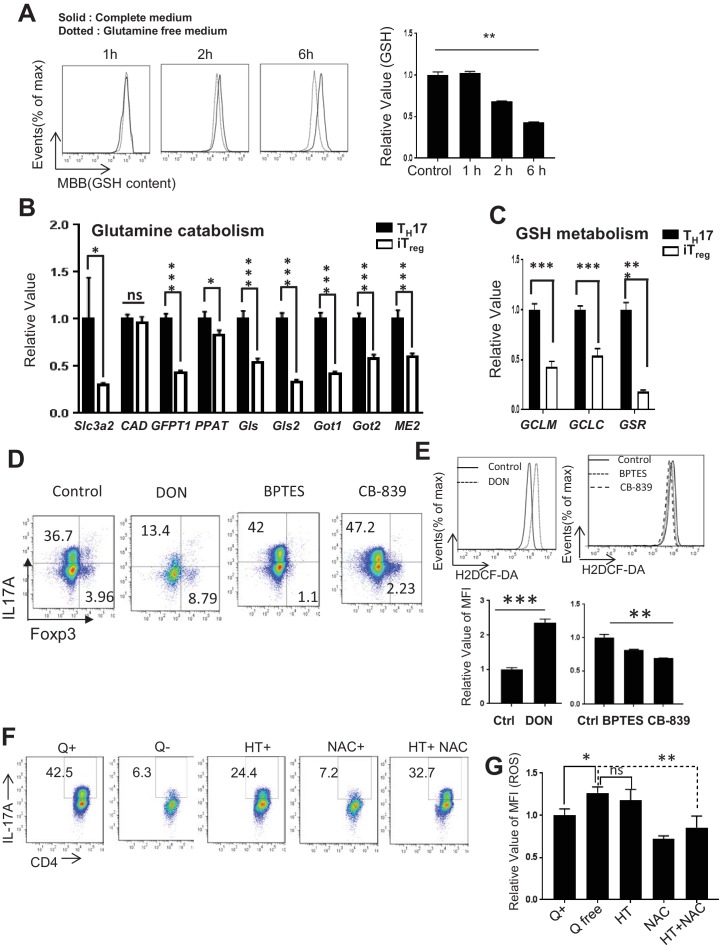
Glutamine catabolism is required for driving T_H_17 and iT_reg_ cell differentiation. (**A**) Naive CD4 +T cells from C57BL/6 mice were differentiated with T_H_17 cell -inducing conditions for 5 days in complete medium and cells were washed and cultured in Q-free medium as indicated time followed by the measurement of GSH. (**B–C**) RNAs were isolated from cells differentiated under T_H_17 or iT_reg_-inducing conditions for 3–5 days, and used for real-time qPCR analyses of indicated metabolic genes. Expression levels in T_H_17 group were set to 1. (**D**) Naive CD4^+^ T cells from C57BL/6 mice were differentiated in presence of 30 µM DON, 25 µM BPTES and 25 µM CB-839 under T_H_17 cell -inducing conditions for 5 days, followed by intracellular staining of IL-17 and Foxp3 (**E**) ROS production by measuring the DCF-fluorescence intensity and the data was represented by histogram (upper panel) and relative intensity by bar graph (lower panel). (**F**) Naive CD4^+^ T cells from C57BL/6 mice were differentiated in presence and absence of glutamine and 5 mM NAC, 100 μM hypoxanthine and 16 μM thymidine (HT) and in combination of HT and NAC under T_H_17 cell-inducing conditions for 5 days, followed by intracellular staining of IL-17 (**G**). Bar graph shows the relative value of mean fluorescence intensity of ROS measurement. Data represent the mean ±S.D of two-three independent experiments. 10.7554/eLife.36158.022Figure 6—figure supplement 1—source data 1.Source data for A, B, C, E and G.

**Figure 6—figure supplement 2.**
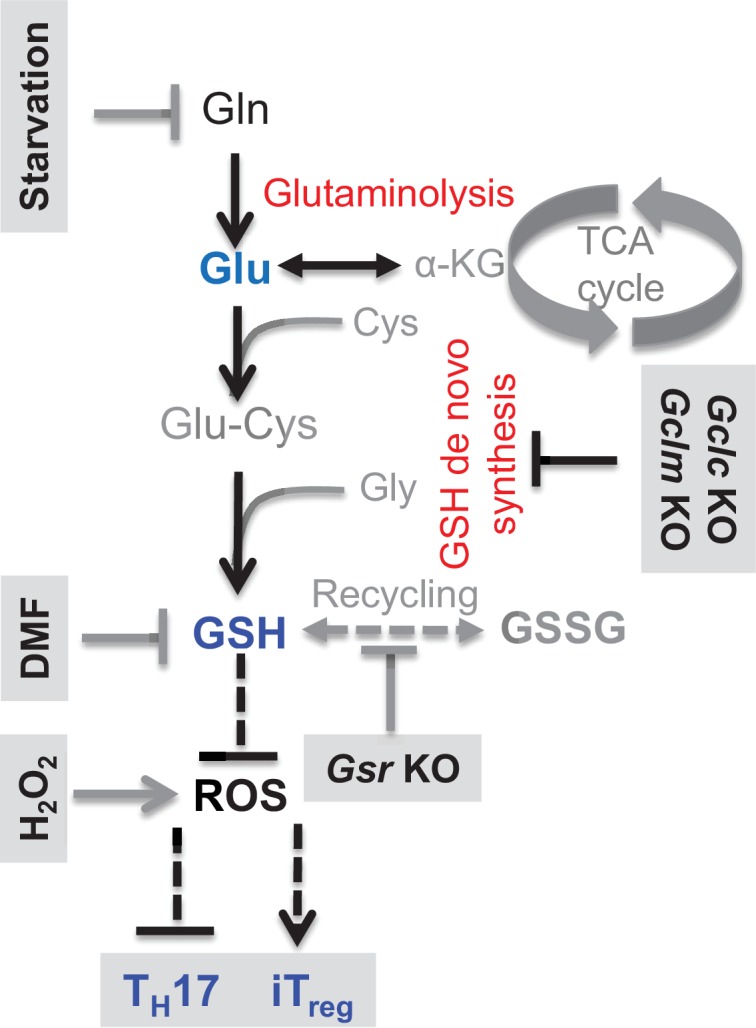
Glutamine catabolism coordinates with GSH metabolism in modulating ROS homeostasis and T cell differentiation. Modulation of GSH biosynthesis and ROS production in directing T cell differentiation.

## Discussion

A robust T cell-mediated adaptive immune response results from the clonal expansion of antigen-specific T cells and subsequent differentiation into diverse functional subsets to fine-tune responses against challenge. Both the cellular proliferation during expansion and the cytokine production associated with differentiation exert high bioenergetic and biosynthetic demand on T cells. Accordingly, rapidly evolving pathogens often impose selective pressures on the regulation of central metabolic pathways that fuel cell proliferation and differentiation, allowing T cells to maintain homeostasis while remaining ready to mount rapid responses under diverse metabolic and immune conditions. The inability to accommodate the metabolic and bioenergetic demands of T cell proliferation and differentiation can impair the proper development and function of T cells. Beyond this, the availability of specific metabolites, and the pathways that process them, interconnect with signaling events in the cell to orchestrate metabolic checkpoints which influence T cell activation, differentiation, and immune function (Wang and Green, 2012; Bensinger and Tontonoz, 2008; Gerriets and Rathmell, 2012; Pearce et al., 2013; Chi, 2012; Powell and Delgoffe, 2010; Michalek and Rathmell, 2010; Ho et al., 2015). Here, we found that glucose plays an indispensable role in driving T_H_17 and iT_reg_ cell differentiation, while glutamine is only required for T_H_17 cell differentiation. Glutamine catabolism is coupled with de novo GSH synthesis and is preferentially elevated during T_H_17 but not iT_reg_ cell differentiation, suggesting that different T cell lineages ‘wire’ metabolism differently to support their development and function. Previous studies have suggested that engagement of the transcription factor hypoxia-inducible factor 1α (HIF1α) coordinates glycolysis and T cell signaling cascades to regulate the differentiation of T_H_17 and iTreg cells (Shi et al., 2011; Dang et al., 2011). While the high rate of glutamine catabolism ensures the capacity to supply glutamate, glycolysis provides ATP and biosynthetic precursors for glycine, fulfilling the needs of de novo synthesis of GSH during T cell differentiation. Our results using genetic modulation of metabolic enzymes suggest that de novo synthesis of GSH but not recycling from GSSG is required for fine-tuning ROS and directing the differentiation of T_H_17 and T_reg_ cells. We further found that dimethyl fumarate, a FDA approved drug (BG-12/Tecfidera) for multiple sclerosis, suppresses T_H_17 differentiation by augmenting intracellular ROS. While previous studies clearly demonstrate that activation-induced metabolic reprogramming is required for driving T cell growth and proliferation, our studies shed light on the complex utilization of the glutamine catabolic pathway and implicate ROS as essential metabolic signals that dictate T cell lineage engagement.

Prokaryotic and eukaryotic cells have evolved to maintain reducing intracellular conditions by generating reducing equivalents, NADPH and GSH, enabling cells to fine-tune the ROS levels that are required for fulfilling vital cellular functions. Accordingly, oxidative stress occurs if the balance between ROS production and antioxidant capacity is disturbed, thereby leading to the accumulation of oxidized biomolecules. T cell receptor (TCR) engagement induces a rapid generation of mitochondrial ROS from OXPHOS and cytoplasmic ROS from NADPH oxidases (NOXs), a family of plasma membrane associated oxidases (Sena et al., 2013; Kamiński et al., 2012; Jackson et al., 2004). T cells with reduced production of mitochondrial ROS display impaired production of interleukin 2 (IL-2) and antigen-specific proliferation, indicating an essential signaling role for mitochondrial ROS in driving optimal TCR signaling (Sena et al., 2013; Kamiński et al., 2012). Beyond that, NOX-dependent ROS also plays a role in modulating TCR signaling and T cell differentiation. T cells with low levels of ROS due to NOX deficiency are skewed toward a T_H_17 phenotype (Jackson et al., 2004; Purushothaman and Sarin, 2009; Padgett and Tse, 2016; Tse et al., 2010). Our studies suggest that de novo synthesis of GSH is essential for fine-tuning ROS levels in T cells and also indicate the differential requirements for the level of ROS in driving T cell lineage engagement toward T_H_17 or iT_reg_ cells. Understanding the metabolic process of GSH synthesis and ROS generation during T cell differentiation may also impact the development of safer and more effective therapies for autoimmune and inflammation diseases. Dimethyl fumarate (DMF) is a cellular permeable analog of fumarate and the key active ingredient of BG-12/TECFIDERA and FUMADERM, a first-line oral treatment for relapsing multiple sclerosis (CONFIRM Study Investigators et al., 2012; ALLEGRO Study Group et al., 2012). However, the cellular and molecular mechanisms of DMF therapy remain largely elusive. DMF has been suggested to impact a plethora of cellular and molecular targets, including Nrf2 and GPCRs in T cells, dendritic cells (DCs), endothelial cells, and neurons (Blewett et al., 2016; Schulze-Topphoff et al., 2016; Wang et al., 2015; Chen et al., 2014; Peng et al., 2012; Linker and Gold, 2013; Duffy et al., 1998; Gill and Kolson, 2013). Consistent with the studies showing that DMF can neutralize GSH in vitro (Zheng et al., 2015; Sullivan et al., 2013), our data indicate that the ability of DMF to augment ROS and suppress T_H_17 cell differentiation contributes to its therapeutic effect.

We found that DMF treatment induced a compensatory NRF2-mediated anti-oxidative response and NRF2 deficiency exacerbated the ROS-producing effects of DMF in T_H_17 cells. Also, NRF2 and its target genes were highly upregulated in T_H_17 cells, suggesting that T_H_17 cells preferentially maintain a low degree of oxidative stress partially by engaging the NRF2 signaling pathway. In addition to GSH production and consumption, NRF2 controls thioredoxin (TXN) production and consumption through transcriptional regulation of its target genes including thioredoxin reductase 1 (TXNRD1). Both GSH and TXN are important anti-oxidation systems and appear functionally redundant in most organisms (Couto et al., 2016; Mustacich and Powis, 2000; Lei et al., 2016; Lu and Holmgren, 2014). However, recent studies implicate the presence of a dynamic cross-talk between these two systems. Glutaredoxin (GRX)-GSH can reduce oxidized TXN in the absence of thioredoxin reductase. Conversely, TXN-TXNRD can also function as an alternative system to reduce GSSG to GSH (Du et al., 2012; Tan et al., 2010; Johansson et al., 2004; Hanschmann et al., 2010). Collectively, these data indicate that the TXN and GSH systems can backup for each other under certain conditions. Consistent with the overlapping function of the GSH and TXN system, the inhibition of TXNRD rendered cancer cells susceptible to the depletion of GSH (Mandal et al., 2010; Lu et al., 2007; Wang et al., 2012). Although our data suggest that the de novo synthesis of GSH is sufficient to maintain ROS homeostasis in the absence of Gsr dependent GSSG-GSH recycling, it is still conceivable that thioredoxin reductase may partially compensate for the loss of Gsr by backing up the recycle of GSSG to GSH in T cells. It has been shown that TXN can be secreted by CD4^+^ T cells and may modulate the expression of T cell surface receptor and proliferation (Rosen et al., 1995; Wakasugi et al., 1990; Matthias et al., 2002; Tagaya et al., 1990). As such, we envision that the combination of DMF and pharmacological approaches that target TXN system may represent a more effective strategy than DMF alone for treating T cell-mediated inflammation and autoimmune disease.

## Materials and methods

**Key resources table:** 

Reagent type (species) or resource	Designation	Source or reference	Identifiers	Additional information
Strain, strain background (Mus musculus)	C57BL/6 (B6) mice		Taconic	
Strain, strain background (Mus musculus)	C3H/HeN		Envigo	
Genetic reagent (Mus musculus)	*CD4-Cre* *Gclc^flox/flox^*	PMID:23226398		
Genetic reagent (Mus musculus)	*Gclm-*KO	PMID:12384496		
Genetic reagent (Mus musculus)	*ROSA26CreERT2*	RRID: IMSR_JAX:008463	The Jackson Laboratory	
Genetic reagent (Mus musculus)	*GSR-*KO	PMID: 10218442		
Antibody	Mouse anti- CD3 mAb	Cat. #:BE0001-1, RRID:AB_1107634	BioXcell	
Antibody	Mouse anti- CD28 mAb	Cat. #BE0015-1 RRID:AB_1107624	BioXcell	
Antibody	Mouse anti -IL2 mAb	Cat.#BE0043 RRID::AB_1107702	BioXcell	
Antibody	Mouse anti- IL4 mAb	Cat. #BE0045 RRID:AB_1107707	BioXcell	
Antibody	Mouse anti- IFNγ mAb	Cat. #BE0055 RRID:AB_1107694	BioXcell	
Antibody	Anti mouse CD4-FITC	Cat. #11–0042 RRID:AB_464897	eBioscience	(1:200)
Antibody	Anti mouse CD4-APC	Cat. #17-0041-81 RRID:AB_469319	eBioscience	(1:200)
Antibody	Anti mouse CD8-APC-Cy7	Cat. #100714 RRID:AB_312753	Biolegend	(1:200)
Antibody	Anti mouse Foxp3-APC	Cat. # RRID:AB_469456	eBioscience	(1:200)
Antibody	Anti mouse IL-17A-PECy7	Cat. #25-7177-82 RRID:AB_10732356	eBioscience	(1:200)
Antibody	Anti GCLC antibody (rabbit monoclonal)	Cat. #ab190685 RRID:AB_10975474	Abcam	WB (1:1000)
Antibody	Anti GCLM antibody (rabbit monoclonal)	Cat. #ab124827 RRID:AB_10975474	Abcam	WB (1:1000)
Antibody	anti-mouse CD25 -PE	Cat. #101904 RRID:AB_312847	Biolegend	(1:200)
Antibody	anti-mouse CD69-PECy7	Cat. #552879 RRID:AB_394508	BD Bioscience	(1:200)
Antibody	Anti mouse monoclonal CD3	Cat. #sc-101442 RRID:AB_1120355	Santa Cruz	IHC (1:50)
Antibody	Anti mouse monoclonal galectin-3	Cat. #sc-32790, RRID:AB_627657	Santa Cruz	IHC (1:50)
Peptide, recombinant protein	MOG35-55 peptide	synthesized and HPLC-purified	St. Jude Hartwell Center for Biotechnology	
Peptide, recombinant protein	Recombinant mouse IL-6	216–16	Peprotech	
Peptide, recombinant protein	Recombinant human TGFb	100–21 c	Peprotech	
Peptide, recombinant protein	Recombinant human or mouse IL-2	200–02	Peprotech	
Commercial assay or kit	Foxp3/Transcription Factor Staining Buffer Set	00-5523-00	e-Bioscience	
Commercial assay or kit	Naive CD4 + T cell isolation kit,mouse	5160725186	Miltenyi Biotec	
Commercial assay or kit	CD45R(B220) microbeads, mouse	5150309030	Miltenyi Biotec	
Commercial assay or kit	ABC kit	PK-7200	Vector laboratories	
Commercial assay or kit	MojoSort Mouse naive CD4 T Cell Isolation Kit	480031	Biolegend	
Chemical compound, drug	Diethly Fumerate	Sigma Aldrich	D95654	
Chemical compound, drug	N-Acetyl-L-cysteine	Sigma-Aldrich	A7250	
Chemical compound, drug	Tamofixen	Sigma-Aldrich	T5648	
Chemical compound, drug	4-hydroxytamoxifen	Sigma	H7904	
Chemical compound, drug	Dimethy a-keto glutarate/aKG	Sigma-Aldrich	34963–1	
Chemical compound, drug	Hypoxathine	Sigma-Aldrich	H9377	
Chemical compound, drug	Thymidine	Sigma	T9250	
Chemical compound, drug	H2O2	Sigma-Aldrich	7722-84-1	
Chemical compound, drug	carboxyfluorescein diacetate succinimidyl ester(CFSE)	Invitrogen	C1157	
Chemical compound, drug	DM-H2DCFDA	Invitrogen	C6827	
Chemical compound, drug	DAB	Vector Laboratories	SK-4100	
Chemical compound, drug	Monobromobimane	Invitrogen	M1378	
Chemical compound, drug	7-amino- actinomycin D(7AAD)	Biolegend	420404	
Chemical compound, drug	Pertussis toxin	181	List Biological Laboratories	
Chemical compound, drug	Mycobacterium tuberculosum	231141	Difco	
Chemical compound, drug	Incomplete Freund’s Adjuvant	263910	Difco	
Chemical compound, drug	[U-14C]-glutamine	MC 1124	Moravek	
Chemical compound, drug	[2–14C]-pyruvate	ARC 0222	American Radiolabeled Chemicals	
Chemical compound, drug	Cell Stimulation Cocktail (plus protein transport inhibitors) (500X)	00-4975-93	eBioscience	
Chemical compound, drug	Iscove's Modified Dulbecco's Media - Glucose free conditional medium	ME17058P1	Thermo Fisher Scientific	
Chemical compound, drug	Iscove's Modified Dulbecco's Media - without L-glutamine	12–726 f	Lonza	
Chemical compound, drug	RPMI 1640 Medium, No Glucose	11-879-020	Gibco	
Chemical compound, drug	Hyclone RPMI 1640 Medium, no glutamine	sh30096.10	Thermo Fisher Scientific	
Chemical compound, drug	U-13C6-glutamine	CNLM-1275–0.1	Cambridge Isotope Lab	
Chemical compound, drug	6-Diazo-5-oxo-L -norleucine	D2141-5MG	Sigma-aldrich	
Chemical compound, drug	Bis-2- (5-phenylacetamido -1,3,4-thiadiazol-2-yl) ethyl sulfide (BPTES)	SML0601	Sigma-aldrich	
Chemical compound, drug	CB-839	22038	Cayman	
Software, algorithm	Graphpad Prism		RRID:SCR_002798	
Software, algorithm	FlowJo		RRID:SCR_008520	

### Mice

*Gsr-*KO mice are on C3H/HeN background and *ROSA26CreERT2, CD4-Cre, Gclm-*KO, and *Gclc^flox/flox^* are on the C57BL/6 background and were previously described (Wang et al., 2011; Chen et al., 2007; Yang et al., 2002; Rogers et al., 2004; Pretsch, 1999; Yan et al., 2012) C57BL/6 mice were purchased from Envigo (formly Harlan). Mice at 8–12 weeks of age were used in the experiment and were kept in specific pathogen-free conditions within the Animal Resource Center at the Research Institute at Nationwide Children’s Hospital or St. Jude Children's, Research Hospital. Animal protocols were approved by the Institutional Animal Care and Use Committee of the Research Institute at Nationwide Children’s Hospital or St. Jude Children's Research Hospital.

### Flow cytometry

For analysis of surface markers, cells were stained in PBS containing 2% (wt/vol) BSA and the appropriate antibodies from eBioscience. Foxp3 staining was performed according to the manufacturer's instructions (eBioscience). For IL-17A intracellular cytokine staining, T cells were stimulated for 4-5 h with phorbol 12-myristate 13-acetate (PMA) and ionomycin in the presence of monensin before being stained according to the manufacturer's instructions (BD Bioscience). For ROS measurement, cells were cultured in fresh serum-free IMDM media containing 5 μM H_2_DCF-DA (BD Bioscience) for 30 min at 37°C before being washed and resuspended with serum-free IMDM media. The fluorescence intensity was measured by flow cytometry. For GSH measurement, cells were cultured in PBS (1%FBS) containing 50μM monobromobimane (Biochemika) for 10 min at 37°C before being washed and resuspended with PBS. The fluorescence at 450/50 nm (blue spectra) was measured by flow cytometry (Cossarizza et al., 2009). Flow cytometry data were acquired on Novocyte (ACEA Biosciences) or LSRII (Becton Dickinson) and were analyzed with FlowJo software (TreeStar).

### Cell purification and culture

Total T cells or naive CD4^+^ T cells were enriched from spleens and lymph nodes by negative selection using MACS systems (Miltenyi Biotec, Auburn, CA) following the manufacturer’s instructions. Freshly isolated total T cells with 75-80% CD3 positivity were either maintained in culture media with 5ng/ml IL7 or were stimulated with IL-2 (100U/ml) and plate-bound anti-CD3 (clone 145-2C11) and anti-CD28 (clone 37.51). Plates were pre-coated with 2 μg/ml antibodies overnight at 4°C. Cells were cultured in RPMI 1640 media supplemented with 10% (v/v) heat-inactivated fetal bovine serum (FBS), 2 mM L-glutamine, 0.05 mM *2-mercaptoethanol*, 100 units/ml penicillin and 100 μg/ml streptomycin at 37 °C in 5% CO2. For CFSE dilution analysis, cells were pre-incubated for 10 min in 4 μM CFSE (Invitrogen) in PBS plus 5% FBS before culture. For iT_reg_ cell differentiation, 0.5 x10^6^ naive CD4^+^ T cells were stained with 4 μM CFSE and cultured with 100 U/ml IL-2, 5ng/ml TGF-β and 20ng/ml IL-6 in 0.5ml RPMI-1640 media (containing 10% (v/v) heat-inactivated fetal bovine serum (FBS), 2 mM L-glutamine, 0.05 mM *2-mercaptoethanol*, 100 units/ml penicillin and 100 μg/ml streptomycin) in 48-well tissue culture plate that was pre-coated with 10µg/ml anti-CD3 and 10µg/ml anti-CD28 overnight at 4°C . For T_H_17 conditions, 0.5 x10^6^ naive CD4^+^ T cells and 5 x10^6^ irradiated splenocytes (artificial APC) were cultured with 2 μg/ml anti-CD3 (2C11; Bio X Cell), 2 μg/ml anti-CD28 (37.51; Bio X Cell), 8 μg/ml anti–IL-2, 8 μg/ml anti–IL-4, 8 μg/ml anti–IFN-γ, 2 ng/ml TGF-β, and 20-50ng/ml IL-6 in 1ml IMDM media (containing 15% (v/v) heat-inactivated fetal bovine serum (FBS), 2 mM L-glutamine, 0.05 mM *2-mercaptoethanol*, 100 units/ml penicillin and 100 μg/ml streptomycin) in 24-well tissue culture plate). For metabolic starvation experiment, Glucose or Glutamine-free IMDM and RPMI-1640 medium was supplemented with 10% (v/v) heat-inactivated dialyzed fetal bovine serum (DFBS). For T_H_17 rescue experiments, 0.5 x10^6^ naive CD4+ T cells were activated in complete IMDM medium. After 24 h cells were switched to conditional glutamine-free IMDM medium for 4 days. DFBS was made dialyzing against 100 volumes of distilled water (six changes in three days) using Slide-ALyzer G2 dialysis cassettes with cut-through MW size 2K (ThermoFisher Scientific) at 4⁰C.

### qPCR and immunoblot analysis.

Total RNA was isolated using the RNeasy Mini Kit (Qiagen) and was reverse transcribed using random hexamer and M-MLV Reverse Transcriptase (Invitrogen). SYBR green-based quantitative RT-PCR was performed using the Applied Biosystems 7900 Real Time PCR System. The relative gene expression was determined by the comparative *C*_T_ method also referred to as the 2^−ΔΔ*C*^_T_ method. The data were presented as the fold change in gene expression normalized to an internal reference gene (beta2-microglobulin) and relative to the control (the first sample in the group). Fold change=2^−ΔΔ*C*^_T_=[(*C*_Tgene of interst_- *C*_Tinternal reference_)]sample A-=[(*C*_Tgene of interst_- *C*_Tinternal reference_)]sample B. Samples for each experimental condition were run in triplicated PCR reactions. Primer sequences were obtained from Primer Bank (Spandidos et al., 2010). Primer sequences are listed in Supplementary file 1. Cell extracts were prepared and immunoblotted as previously described (Wang et al., 2011).

### MOG immunization and EAE

Mice were immunized with 100 μg of myelin oligodendrocyte glycoprotein (MOG)_35–55_ peptide in CFA (Difco) with 500 μg of Mycobacterium tuberculosis (Difco). Mice were i.p. injected 200 ng of pertussis toxin (List Biological,#181) on the day of immunization and 2 days later, as described (Kang et al., 2010). The mice were observed daily for clinical signs and scored as described previously (Shi et al., 2011).

### Histopathology and immunohistochemistry

Mice were euthanized and then were perfused with 25 ml PBS with 2 mM EDTA by heart puncture to remove blood from internal organs. Spinal cords were taken out and fixed by immersion with 10% neutral buffered formalin solution and decalcified. Spinal column was divided into cervical, thoracic and lumbar, and then was embedded in paraffin, sectioned, and stained with standard histological methods for hematoxylin and eosin (H and E). Immunohistochemistry were performed on serial histological sections according to standard protocols using anti-Mac2 and anti-CD3 (1:50, Santa Cruz). Appropriate horseradish peroxidase (HRP)-conjugated secondary antibodies were used and detected using 3,3’-diaminobenzidine tetrahydrochloride (DAB). Slides were counterstained with hematoxylin. Microscopy images were taken using Zeiss Axio Scope A1.

### Metabolic activity analysis

Glutamine oxidation activity was determined by the rate of ^14^CO_2_ released from [U-^14^C]-glutamine (Brand et al., 1984). In brief, one-five million T cells were suspended in 0.5 ml fresh media. To facilitate the collection of ^14^CO_2_, cells were dispensed into 7 ml glass vials (TS-13028, Thermo) with a PCR tube containing 50 μl 0.2M KOH glued on the sidewall. After adding 0.5 μci [U-^14^C]-glutamine, the vials were capped using a screw cap with rubber septum (TS-12713, Thermo). The assay was stopped 2 hr later by injection of 100 μl 5N HCL and the vials were kept at room temperate overnight to trap the ^14^CO_2_. The 50 μl KOH in the PCR tube was then transferred to scintillation vials containing 10 ml scintillation solution for counting. A cell-free sample containing 0.5 μci [U-^14^C]-glutamine was included as a background control.

Pyruvate oxidation activity was determined by the rate of ^14^CO_2_ released from [2-^14^C]-pyruvate (Willems et al., 1978). In brief, one to five million T cells were suspended in 0.5 ml fresh T cell media. To facilitate the collection of ^14^CO_2_, cells were dispensed into 7 ml glass vials (TS-13028, Thermo) with a PCR tube containing 50 μl 0.2M KOH glued on the sidewall. After adding 0.5 μci [2-^14^C]-pyruvate, the vials were capped using a screw cap with rubber septum (TS-12713, Thermo). The assay was stopped 2 hr later by injection of 100 μl 5N HCL and the vials were kept at room temperate overnight to trap the ^14^CO_2_. The 50 μl KOH in PCR tube was then transferred to scintillation vials containing 10 ml scintillation solution for counting. A cell-free sample containing 0.5 μci [2-^14^C]-pyruvate was included as a background control.

### Metabolite extraction and analysis by ion chromatography-ultra high resolution-Fourier transform mass spectrometry (IC-UHR-FTMS)

Cells were cultured in glutamine-free media with 2 mM ^13^C_5_^15^N_2_-glutamine (Cambridge Isotope Laboratories) for 24 hr at 37°C and were then washed three times in cold PBS before snap freezing. The frozen cell pellets were homogenized in 60% cold CH3CN in a ball mill (Precellys- 24, Bertin Technologies) for denaturing proteins and optimizing extraction. Polar metabolites were extracted by the solvent partitioning method with a final CH3CN:H2O:CHCl3 (2:1.5:1, v/v) ratio, as described previously (Fan et al., 2012). The polar extracts were lyophilized before reconstitution in nanopure water and analysis on a Dionex ICS-5000 +ion chromatography interfaced to a Thermo Fusion Orbitrap Tribrid mass spectrometer (Thermo Fisher Scientific) as previously described (Fan et al., 2016) using a *m/z* scan range of 80–700. Peak areas were integrated and exported to Excel via the Thermo TraceFinder (version 3.3) software package before natural abundance correction (Moseley, 2010). The isotopologue distributions of metabolites were calculated as the mole fractions as previously described (Lane et al., 2008). The number of moles of each metabolite was determined by calibrating the natural abundance-corrected signal against that of authentic external standards. The amount was normalized to the amount of extracted protein, and is reported in µmol/g protein. Metabolome quantification of GSH and GSSG were determined by CE-MS that was carried out through a facility service at Human Metabolome Technology Inc., Tsuruoka, Japan.

### Statistical analysis

*P* values were calculated with Student's *t*-test all experiment except EAE experiments, where two way anova test was performed. *P* values smaller than 0.05 were considered significant, with p-values<0.05, p-values<0.01, and p-values<0.001 indicated as *, **, and ***, respectively.

## Additional files

Supplementary file 1. List of primer sequences used for RT-PCR analysis.

Transparent reporting form

### Data availability

All data generated or analysed during this study are included in the manuscript and supporting files.
